# Seasonal deviation effects foliar endophyte assemblage and diversity in *Asparagus racemosus* and *Hemidesmus indicus*

**DOI:** 10.1186/s12898-018-0211-y

**Published:** 2018-12-04

**Authors:** Riyaz Ahmad Rather, Vijayalakshmi Srinivasan, Mumtaz Anwar

**Affiliations:** 1 Department of Biotechnology, School of Natural and Computational Science, Wachemo University, Hossana, Ethiopia; 2 Department of Biotechnology, VELS University, Chennai, India; 3 Department of Pharmacology, College of Medicine, University of Illinois, Chicago, USA

**Keywords:** Fungal endophyte, Relative colonization density, *Asparagus racemosus*, Mycelia, *Hemidesmus indicus* and *Acremonium strictum*

## Abstract

**Background:**

Fungal endophytes are the living symbionts which cause no apparent damage to the host tissue. The distribution pattern of these endophytes within a host plant is mediated by environmental factors. This study was carried out to explore the fungal endophyte community and their distribution pattern in *Asparagus racemosus* and *Hemidesmus indicus* growing in the study area.

**Results:**

Foliar endophytes were isolated for 2 years from *A. racemosus and H. indicus* at four different seasons (June–August, September–November, December–February, March–May). A total of 5400 (675/season/year) leaf segments harbored 38 fungal species belonging to 17 genera, 12 miscellaneous mycelia sterile from 968 isolates and 13 had yeast like growth. In *A. racemosus*, *Acremonium strictum* and *Phomopsis* sp.1, were dominant with overall relative colonization densities (RCD) of 7.11% and 5.44% respectively, followed by *Colletotrichum* sp.3 and *Colletotrichum* sp.1 of 4.89% and 4.83% respectively. In *H. indicus* the dominant species was *A. strictum* having higher overall RCD of 5.06%, followed by *Fusarium moniliforme* and *Colletotrichum* sp.2 with RCD of 3.83% and 3%, respectively. Further the overall colonization and isolation rates were higher during the wet periods (September–November) in both *A. racemosus* (92.22% and 95.11%) and *H. indicus* (82% and 77.11%).

**Conclusion:**

Study samples treated with 0.2% HgCl_2_ and 75% EtOH for 30 s and 1 min, respectively, confirmed most favorable method of isolation of the endophytes. Owing to high mean isolation and colonization rates, September–November season proved to be the optimal season for endophyte isolation in both the study plants. Assessing the bioactive potential of these endophytes, may lead to the isolation of novel natural products and metabolites.

## Background

Endophytes are highly diverse microorganisms that live as symbionts within plant tissues and usually remain asymptomatic [1]. These symbiotic endophytes serve a tremendous source of secondary metabolites of industrial, agricultural and therapeutic use [2–5]. The role of endophytes in the biotransformation process illustrates their importance to produce chemical changes in non-biodegradable substances [6]. Endophytic fungal communities, their diversity, and distribution patterns have been explored in leaves of tropical forest trees [7]. Arnold and Lutzoni [8] cited tropical areas as better hotspots to explore the hyper-diversity of leaf endophytes. Endophytic fungi were reported from plants under various environmental conditions, like tropic, temperate, xerophytic and aquatic niches [9–12]. Numerous studies suggest that the host-endophyte relationship is variable and host non-specific, implying that a single endophyte can invade wide host range [13]. Therefore, endophyte can be isolated from diverse plants bearing different classification and growing under divergent ecological or geographical niches. Climate change may alter the degree of mutualism between plant and fungi that even changes the efficacy of transmission of the endophyte from one plant to another [14]. Recent studies suggest that fungal diversity is richer in the tropics than in the temperate regions and one can witness temporal changes among endophytic fungi [15]. Most mycologist suggests that fungal diversity crest in tropical areas where woody angiosperm diversity is higher [16]. The endophyte distribution within the plant is controlled by genes of both the plant and endophyte and modulated by the environment. Different parameters of the environment like rainfall, temperature, humidity, terrain, or season may play an important role in the distribution pattern of endophytes within a host plant [17].

Plants are known to harbor endophytic fungi that are believed to be associated with the production of pharmaceutical products [18]. Endophytic fungi have been isolated from *Asparagus racemosus* (Wild.) and *Hemidesmus indicus* (Linn.) [19, 20]. *A. racemosus* is a climbing undershrub found in tropical and subtropical Indian subcontinent having tremendous medicinal properties. *H. indicus* belongs to the family Asclepiadaceae. It is a semi-erect shrub with slender stem thickened at the nodes. These two plants have a large diversity in tropical areas and, therefore, are expected to possess high endophyte diversity [21]. Hence, major efforts are taken to isolate and characterize endophytes from plants that bear ethnobotanical history. The aim of the present study was to explore the fungal endophyte community of *A*. *racemosus* and *H*. *indicus* and to check the seasonal deviation effect on the isolation and the distribution pattern of fungal endophytes within the study plants.

## Methods

### Collection of plant samples

The healthy plant samples of *A. racemosus* and *H. indicus* were procured from Irula Tribe Women’s Welfare Society, Thandarai, Chennai, India (12°39′32″–12.6°39′32″N:79.6° 2′45″–80°2′45″E). After procurement, the plants were identified by the taxonomist and further utilized in the study. The institutional guidelines were strictly followed while acquiring and processing the study plants. The matured leaf samples of both the plants were collected and placed in separate self-sealing plastic bags and returned to the laboratory on the same day and kept at 4 °C until the next morning for the isolation of endophytic fungi. The samples were collected every 3 months during a year (June–August, September–November, December–February, March–May) and the collection was repeated for 2 years.

### Isolation of fungal endophytes

Fungal endophytes were isolated by adopting the method of Suryanarayan and Thennarasan [22, 23] with slight modification. Surface sterilization of leaf samples was done by adopting 10 different sterilization methods, to evaluate the best yielding method for endophytic isolation. The efficacy of each method was validated by three different conventional methods viz, by inoculating the surface sterilized sample onto nutrient media, by inoculating 0.5 mL aliquots of water from the last rinsing of sample onto nutrient media and, by plummeting the surface sterilized plant samples of each method into nutrient broth. The different methods are briefly described as follows: In method 1 the leaf samples were treated with 0.1% mercuric chloride (HgCl_2_) and 70% ethanol (EtOH) for 1 min each; method 2 (0.2% HgCl_2_ and 70% EtOH for 35 s and 1 min respectively); method 3 (0.3% HgCl_2_ and 70% EtOH for 25 s and 1 min respectively); method 4 (0.4% HgCl_2_ and 70% EtOH for 15 s and 1 min respectively); method 5 (0.2% HgCl_2_ and 25% EtOH for 30 s and 2 min respectively); method 6 (0.2% HgCl_2_ and 50% EtOH for 30 s and 1.5 min respectively); method 7 (0.2% HgCl_2_ and 75% EtOH for 30 s and 1 min respectively); method 8 (0.2% HgCl_2_ and 99% EtOH for 30 s each); method 9 (4% sodium hypochlorite and 75% EtOH for 2 and 1 min respectively); and method 10 (4% sodium hypochlorite and 99% EtOH for 2 min and 30 s respectively). The method yielding significant (p < 0.05) highest number of endophytes was chosen as a standard method for further isolation procedure. The leaves were thoroughly washed in running sterile water and dried in laminar air flow hood before further processing. Leaf segments of *H. indicus* were cut into 0.5 cm^2^ and needle-like pinnate and spinous leaves of *A. racemosus* were cut straight into 0.5–1 cm due to reduced surface area. Five leaf segments from each plant were inoculated aseptically on petri plates containing potato dextrose agar (PDA) (Difco) amended with chloramphenicol (120 mg/L) and incubated for 21 days at 21 ± 3 °C in a light chamber with a light regimen of 12:12 h light:dark.

### Identification of endophytes

The growing edges of the colonies from the segments were transferred to fresh PDA plates by hyphal tipping and subcultured. For tentative identification, microscopic slides of each endophyte were prepared by staining with lactophenol cotton blue and were examined under a light microscope (Olympus, USA) for colony morphology, conidial ontogeny and spore characteristics. Molecular identification was carried out by the acquisition of internal transcribed spacers (ITS) of ribosomal DNA (rDNA). The ITS regions of the fungi were amplified with the universal ITS-1 (5′TCCGTAGGTGAACCTGCGG3′) and ITS-5 (5′GGAAGTAAAAGTCGTAACAA3′) primers, using polymerase chain reaction (PCR). Each endophyte was cultured separately in potato dextrose broth at 25 °C with constant shaking for 15 days. The fungal mycelia were harvested, freeze-dried and the genomic DNA was extracted by the cetyl trimethylammonium bromide (CTAB) method. Briefly, 750 mg of fungal mycelia was crushed into fine powder and lysed in 15 mL of extraction buffer (50 mM Tris–HCl pH 8.0, 50 mM EDTA, 0.7 M NaCl, 2% cetrimide, 1% SDS), mixed thoroughly and incubated at 65 °C for 40 min with constant shaking. An equal volume of chloroform/isoamyl alcohol (24:1) was added to the lysate and centrifuged at 10,000×*g* for 10 min at 4 °C. The aqueous phase was transferred to a sterile tube; the genomic DNA was precipitated in a 2.5 × volume of chilled isopropanol and centrifuged at 10,000×*g* for 10 min at 4 °C. The resulting pellet was washed thrice with 70% ethanol, air dried and dissolved in 10 μl of sterile DNAase free water. One µL of the undiluted extracted DNA was used in NanoDrop ND-1000 spectrophotometer (NanoDrop Technologies, Wilmington, DE) to check the purity and concentration. The purity of the extracted DNA was based on the A_260_:A_280_ optical density ratio as calculated and DNAase free water was used as a control. The extracted DNA was further subjected to real-time PCR amplification on an ABI 7500 detection system (Applied BioSystems) using power SYBR^®^ Green chemistry. Amplification reactions were set up in a reaction volume of 25 µL which consists of 10 µL power SYBR^®^ Green PCR Master Mix (Applied Biosystems) and 5 µL of template DNA. Primers were used at final concentrations of 300 nM. DNA amplification was carried out in 96 well plates (Applied Biosystems). The PCR conditions used were set as follows: initial denaturation at 95 °C for 10 min followed by 40 cycles of 95 °C for 15 s, 55 °C for 30 s, 72 °C for 45 s, and a final extension at 72 °C for 7 min. Melting curve analysis was carried out at the end of each PCR assay to verify the specificity of the amplified PCR products. The amplified products were sequenced and aligned with the sequences in the GenBank by BLAST-N program to find out the sequence homology with closely related organisms. Endophytes showing complete sequence homology to each other and belonging to the same plant sample were treated as a single isolate.

### Statistical analysis

Measurement of fungal occurrence was established by calculating colonization and isolation rates. The density of colonization was calculated as the percentage of segments infected by one or more isolate(s) from the total number of segments of each tissue plated [24].

$$ {\text{Colonization}}\;{\text{rate}} = \frac{{{\text{Total}}\;{\text{no}}.\;{\text{of}}\;{\text{leaf}}\;{\text{segments}}\;{\text{in}}\;{\text{a}}\;{\text{sample}}\;{\text{yielding}} \ge 1\;{\text{isolates}}}}{{{\text{Total}}\;{\text{no}}.\;{\text{of}}\;{\text{leaf}}\;{\text{segments}}\;{\text{in}}\;{\text{that}}\;{\text{sample}}}} $$

$$ {\text{Isolation}}\;{\text{rate}} = \frac{{{\text{Total}}\;{\text{no}}.\;{\text{of}}\;{\text{isolates}}\;{\text{yielded}}\;{\text{by}}\;{\text{a}}\;{\text{given}}\;{\text{sample}}}}{{{\text{Total}}\;{\text{no}}.\;{\text{of}}\;{\text{leaf}}\;{\text{segments}}\;{\text{in}}\;{\text{that}}\;{\text{sample}}}} $$

Graph Pad Prism version 5.0 (La Jolla, CA, USA) was used for statistical analysis. For each parameter, the mean, standard error of the mean, and range were calculated. Differences were evaluated by t-tests and nonparametric Mann–Whitney test and values of p ˂ 0.05 were considered statistically significant. One way ANOVA was performed to compare the isolation and colonization rates of fungal endophytes of each plant isolated from each season [25].

## Results

The three methods of sterility check employed to confirm the efficacy of each sterilization method used in this study, produced similar results under optimal condition, no bacterial or fungal growth occurred on the control medium, so the surface sterilization was considered absolute and the isolates were deemed as fungal endophytes.

**Fig. 1:**
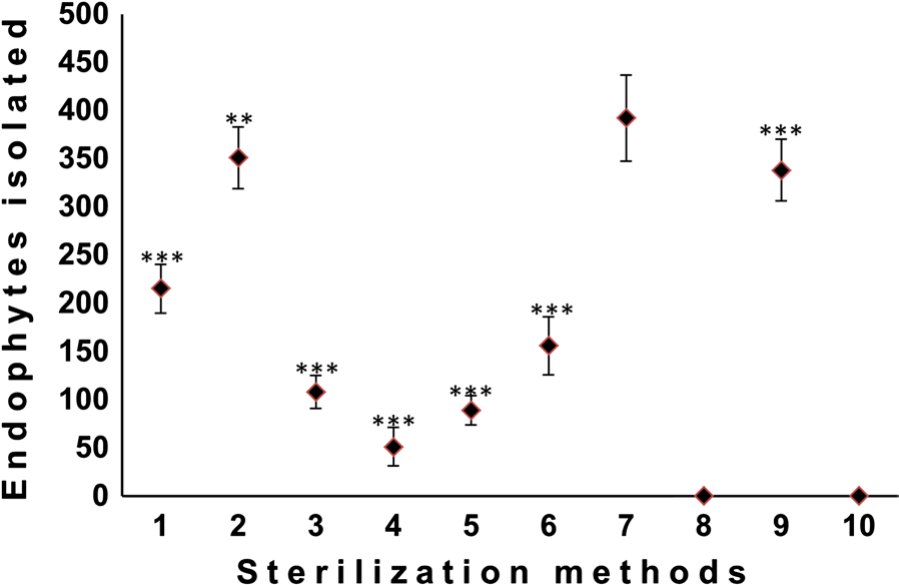
Ten different methods of sterilization and number of fungal endophytes isolated. (* vs method 7); ***p < 0.001, **p < 0.01, *p < 0.05

Among 10 different methods of surface sterilization, method 7 yielded the maximum significant number of endophytes (p < 0.001). Method 2 and 9 yielded the second largest number of endophytes respectively. Though only a few endophytes were isolated by employing method 8, the isolation number of endophytes was non-significant (p > 0.05). Method 10 does not produce any endophyte, indicating that the endophytes within the plant tissue might have been destroyed, though not verified in our study. Moreover, the methods 1, 3, 4, 5 and 6 yielded few non-significant number of endophytes compared to the other method (p > 0.05) (Fig. 1).

The samples which were collected every 3 months during a year (June–August, September–November, December–February, March–May) have different environmental and climatic conditions. The June–August and March–May, periods are hot and dry with an average temperature of 39.8 and 42.3 °C and relative humidity (%) of 62 and 67 respectively. Whereas, the September–November and December–February months are wet with 37.1 and 34.3 °C and relative humidity (%) of 75.6 and 74 respectively.

**Fig. 2:**
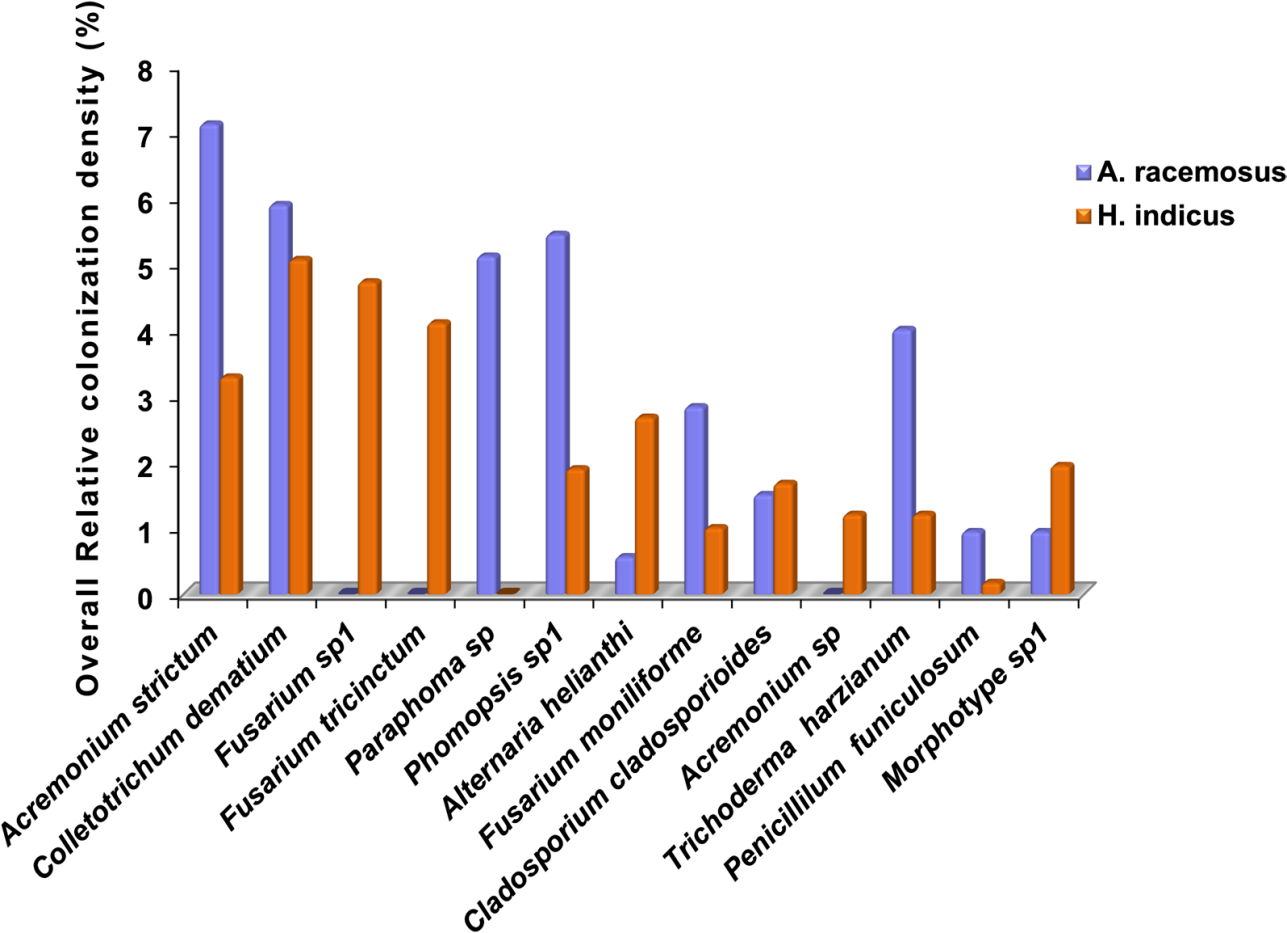
Overall relative colonization density (%) of fungal endophytes from *A. racemosus* and *H. indicus*

**Fig. 3:**
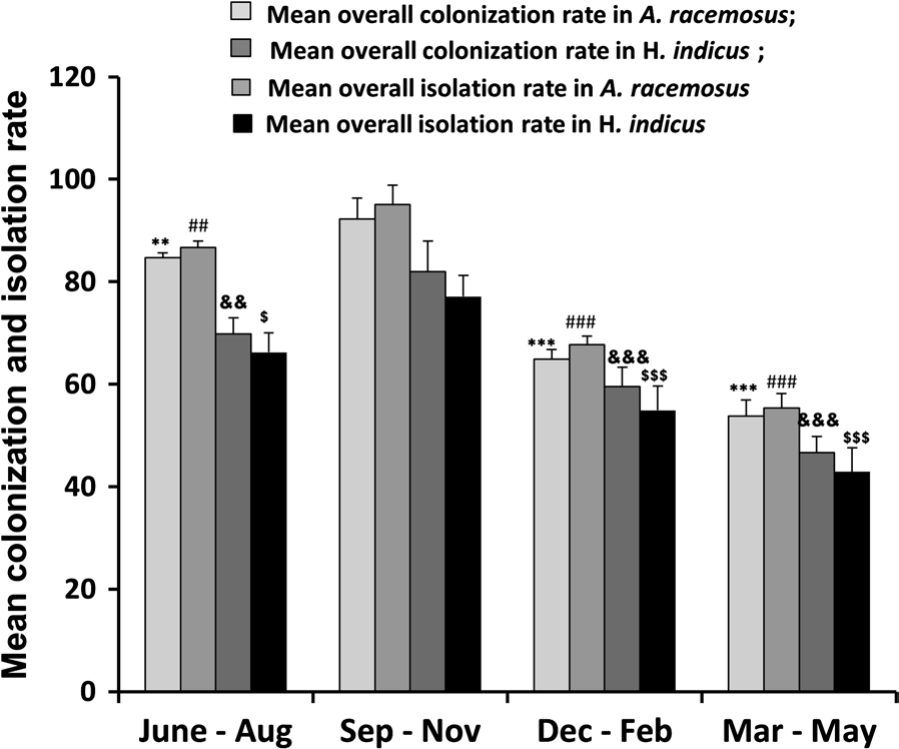
Mean colonization and isolation rates of fungal endophytes of *A. racemosus* and *H. indicus* in different seasons of the year. (* = vs colonization rate in *A. racemosus* in September–November); (^#^ = vs isolation rate in *A. racemosus* in September–November); (^&^ = vs colonization rate in *H. indicus* in September–November); (^$^ = vs isolation rate in *A. racemosus* in September–November), **p < 0.01; ***p < 0.001; ^##^p < 0.01; ^###^p < 0.001; ^&&^p < 0.01; ^&&&^p < 0.001; ^$^p < 0.05; ^$$$^p < 0.001

From 5400 leaf segments (675 leaf segments/season/year), a total of 2457 endophyte isolates belonging to 25 genera were harbored from both the plants. These isolates belonged to Ascomycota (88%) and sterile morphotypes (12%). The genera of ascomycetous fungi belonged to Sordariomycetes (36.3%), Dothidiomycetes (1.6%), Eurotiomycetes (4.5%), Pezizomycetes (4.5%), Saccharomycetes (36.3%). and Mucoromycotina (4.5%). *Colletotrichum*, *Fusarium*, *Alternaria*, *Chaetomium*, *Penicillium*, *Curvularia*, *Acremonium*, *Aspergillus*, and *Cladosporium* were the most abundant and frequently isolated genera from both the plants (Fig. 2). In *A. racemosus*, the dominant species isolated were *Acremonium strictum*, *Colletotrichum dematium*, *Phomopsis* sp.1 and *Paraphoma* sp., with overall RCD 7.11, 5.89, 5.44, and 5.11%, respectively, followed by *Cochliobolus lunatus*, *Phomopsis* sp.2, *Glomerella acutata* and *Trichoderma harzianum* with overall RCD 4.83, 4.39, 4.36, and 4% respectively (Table 1). *Acremonium strictum* was the most recurring endophyte in all the seasons except the June–August season of the second year, with the highest average 12% colonization density in March–May season in both the years (Table 1). *Colletotrichum dematium*, was the second most recurrent endophyte in all the seasons barring December–February season, with 9.89 and 9.3% colonization density in June–August season of the year 1 and 2 respectively (Table 1). Mean colonization rate (%) was significantly (p = 0.023) different between the four seasons with mean colonization rate 84.6, 92.2, 64.8 and 53.7% in June–August, September–November, December–February, and March–May respectively (Table 2, Fig. 3). Similarly, the mean isolation rate (%) of endophytes in *A. racemosus* was 86.6 and 95.1% in June–August and September–November respectively, whereas in December–February and March–May it was just 67.7 and 55.3% respectively (Table 2, Fig. 3). The total fungal species richness associated with *A. racemosus* was 27 with an Evenness (J) of 0.95 and Shannon diversity index (H) was found ~ 2.9. Simpson’s diversity index (D) revealed that higher abundance of fungal species was persistent in *A. racemosus* (D = 0.5) (Table 2).

**Table 1:** Relative colonization density (% RCD) of fungal endophytes isolated from *Asparagus racemosus*

Endophyte	% RCD (year 1)				% RCD (year 2)				Overall % RCD
	June–August	September–November	December–February	March–May	June–August	September–November	December–February	March–May	
*Acremonium strictum*	3.56	6.67	6.67	12.00	–	5.33	10.67	12.00	7.11
*Alternaria helianthi*	–	1.78	–	–	–	0.89	1.78	–	0.56
*Apiosordaria otanii*	4.89	5.33	4.44	–	–	4.00	4.89	–	2.94
*Aspergillus flavus*	–	0.89	–	–	–	0.44	–	2.22	0.44
*Aspergillus niger*	1.33	2.22	–	3.11	3.56	1.78	–	4.00	2.00
*Chaetomium globosum*	–	1.78	0.89	–	–	–	–		0.33
*Cladosporium cladosporioides*	2.22	1.78	–	8.00	–	–	–	–	1.50
*Cochliobolus lunatus*	–	4.89	4.0	9.33	–	4.89	7.11	8.44	4.83
*Colletotrichum dematium*	9.89	8.33	–	7.56	9.3	4.44	–	7.56	5.89
*Colletotrichum lindemuthianum*	7.56	8.89	4.89	–	11.11	6.67	–	–	4.89
*Fusarium moniliforme*	4.44	4.44	1.78	–	8.44	3.56	–	–	2.83
*Fusarium oxysporum*	–	–	–	–	7.11	3.11	4.00	–	1.78
*Geotrichum clavatum*	–	–	–	–	–	4.44	5.33	6.22	2.00
*Geotrichum* sp.	–	–	–	–		4.00	2.22	–	0.78
*Gibberella avenacea*	3.11	0.89	–	0.89	3.11	–	–	–	1.00
*Glomerella acutata*	8.44	6.78	4.44		4.33	6.67	4.22		4.36
*Humicola* sp.	0.44	4.00	1.33	–	–	–	–	–	0.72
*Hypoxylon fragiforme*	–	3.56	2.22	2.22	–	2.67	3.11	1.33	1.89
*Myrothecium verrucaria*	–	–	–	–	6.22	0.44	0.89	–	0.94
*Paraphoma* sp.	5.78	4.89	6.22	–	8.44	7.56	8.00	–	5.11
*Penicillium funiculosum*	2.67	–	–	4.8	–	–	–	–	0.94
*Pestalotiopsis guepinii*	–	6.67	7.56	–	–	–	–		1.78
*Phomopsis* sp.1	11.11	5.78	9.33	–	12.44	4.89	–	–	5.44
*Phomopsis* sp.2	13.78	7.56	8.00	–	–	5.78	–	–	4.39
Morphotype sp.1	1.33	–	–	–	–	6.22	–	–	0.94
*Trichophaea abundans*	4.00	–	–	3.56	4.00	–	–	2.22	1.72
*Trichoderma harzianum*	–	2.67	3.11	5.78	–	2.67	8.44	9.33	4.00

**Table 2:** Diversity indices along with colonization and isolation rates (%) of endophytic fungi isolated from *Asparagus racemosus* and *Hemidesmus indicus* in different seasons

Plant	Colonization and isolation rate (%)	June–August		September–November		December–February		March–May		Shannon (H)	Eveness (J)	Simpson (D)
		Year 1	Year 2	Year 1	Year 2	Year 1	Year 2	Year 1	Year 2			
*Asparagus racemosus*	Colonization rate	85.33	84.00	95.11	89.33	66.22	63.56	56.00	51.56	2.9	0.95	0.5
	Mean colonization rate for individual season	84.6		92.2		64.8		53.7				
	Overall colonization rate	73.8										
	Isolation rate	87.56	85.78	97.78	92.44	68.89	66.6	57.33	53.33			
	Mean isolation rate for individual season	86.6		95.1		67.7		55.3				
	Overall isolation rate	76.2										
*Hemidesmus indicus*	Colonization rate	72.00	67.56	86.22	77.78	62.22	56.89	48.89	44.44	2.7	0.92	0.8
	Mean colonization rate for individual season	69.78		82.00		59.56		46.67				
	Overall colonization rate	64.5										
	Isolation rate	68.89	63.56	80.00	74.22	58.22	51.56	46.22	39.56			
	Mean isolation rate for individual season	66.22		77.11		54.89		42.89				
	Overall isolation rate	60.2

In *H. indicus*, the dominant genera isolate was *Fusarium*, *Penicillium*, *Alternaria*, and *Aspergillus*. *Colletotrichum dematium* was the most dominant species isolated, having overall RCD 5.06%, followed by *Fusarium* sp.1, *Fusarium tricinctum*, and *Acremonium strictum*, with overall RCD 4.72, 4.10 and 3.28% respectively (Table 3, Fig. 2). The least dominant species isolated were *Penicillium oxalicum and Penicillium funiculosum* each with 0.17% overall RCD, followed by *Fusarium redolens*, *Aspergillus fumigatus*, and *Gloeosporium* sp., with 0.33, 0.39 and 0.89% overall RCD (Table 3). The mean colonization rate (%) was considerably variable and significantly (p = 0.012) different between all the four seasons. The absolute percentage of mean colonization rate for season June–August, September–November, December–February, and March–May was 69.78, 82.0, 59.56 and 46.6% respectively (Table 2, Fig. 3). The mean isolation rate (%) of endophytes was observed at 66.22, 77.11, 54.89 and 42.89% in June–August, September–November, December–February, and March–May, respectively (Table 2, Fig. 3). Diversity indices of fungal endophytes varied marginally in *H. indicus*. The Shannon and Simpson’s diversity indices were 2.7 and 0.8 respectively, whereas an Evenness of 0.92 was observed with a maximum of 34 species identified in *H. indicus* (Table 2).

**Table 3:** Relative colonization density (% RCD) of fungal endophytes isolated from *Hemidesmus indicus*

Endophyte	% RCD (year I)				% RCD (year II)				Overall % RCD
	June–August	September–November	December–February	March–May	June–August	September–November	December–February	March–May	
*Acremonium strictum*	2.2	6.67	2.22	5.78	–	5.33	–	6.22	3.28
*Acremonium* sp.	–	3.56	–	6.22	–	–	–	–	1.22
*Alternaria alternata*	–	4.44	4.00	5.33	–	4.44	4.44	–	2.83
*Alternaria helianthi*	–	–	5.78	6.67	–	–	4.00	4.89	2.67
*Alternaria* sp.1	2.67	–		4.89	–	–	4.00	–	1.44
*Aspergillus flavipes*	–	0.44		3.56	–	0.44	0.44	1.78	0.83
*Aspergillus fumigatus*	2.22	0.44	0.44	–	–	–	–	–	0.39
*Chaetomium globosum*	–	1.33	1.33	–	–	0.89	–	–	0.44
*Cladosporium cladosporioides*	–	2.67	0.44	4.89	–	2.22	3.11	–	1.67
*Cladosporium sphaerospermum*	–	–	–	–	3.11	1.78	–	–	0.61
*Cochliobolus lunatus*	5.78	5.33	–	–	5.33	4.89	–	–	2.67
*Colletotrichum dematium*	7.11	5.78	4.44	–	6.67	5.33	5.78	5.33	5.06
*Colletotrichum* sp.	–	6.67	5.33	–	–	6.22	–	5.78	3.00
*Curvularia lunata*	4.89	1.78	1.78	–	4.00	1.78	2.22	–	2.06
*Fusarium moniliforme*	4.00	–	–	4.00	–	–	–		1.00
*Fusarium* sp.1	6.22	6.67	7.30	5.78	–	5.33	2.20	4.22	4.72
*Fusarium oxysporum*	–	–	–	–	–	0.89	1.78	3.11	0.72
*Fusarium redolens*	–	0.89	1.78	–	–	–	–	–	0.33
*Fusarium solani*	5.33	4.00	–	–	5.33	4.44	–	–	2.39
*Fusarium tricinctum*	4.00	3.30	–	4.89	6.22	6.22	4.20	4.00	4.10
*Fusarium* sp.2	4.44	4.00	5.33	–	4.89	–	4.89	–	2.94
*Gibberella moniliformis*	–	3.11	4.00	–	4.89	4.00	4.00	–	2.50
*Gloeosporium* sp.	–	–	–	–	–	3.56	3.56	–	0.89
Morphotype sp.1	4.89	5.33	–	–	4.00	–	–	1.33	1.94
Morphotype sp.2	2.67	3.11	–	–	–	2.22	2.67	–	1.33
*Nigrospora sphaerica*	–	2.67	3.11	–	–	3.11	3.11	4.00	2.00
*Penicillium expansum*	–	–	–	–	2.22	0.44	–	2.22	0.61
*Penicillium funiculosum*	–	–	–	–	–	0.44	0.89	–	0.17
*Penicillium oxalicum*	–	0.89	0.44	–	–	–	–	–	0.17
*Penicillium* sp.	–	4.89	7.11	–	–	4.00	6.67	–	2.83
*Phomopsis* sp.	–	3.56	4.89	–	3.56	3.11	–	–	1.89
*Syncephalastrum racemosum*	5.33	2.67	2.67	–	4.00	2.67	–	–	2.17
*Trichoderma harzianum*	3.56	0.89	0.89	–	3.56	0.89	–	–	1.22

## Discussion

To yield the significant number of endophytes, the sterilization procedure was optimized according to the sample characteristics. The plant sample carries a wide range of epiphytes on its surface, which are the primary source of contamination in endophyte isolation. Hence, to avoid this source of infection, the sample was thoroughly sterilized with the appropriate surface sterilization procedure before inoculating them onto the nutrient medium. An inefficient sterilization procedure with a higher concentration of sterilizing agent and prolonged time of exposure, sometimes can destroy endophytes [26]. In this study, we observed that sterilization method directly affects the number of fungal endophyte isolated. In our study, the survival and contamination percentage of endophytes from the explant didn’t give satisfying results when the concentration of sterilizing agent or treatment duration was either increased or decreased (methods 1, 3, 4, 5 and 6). Leaf samples which were treated with 0.2% HgCl_2_ for 30 s and 75% EtOH for 1 min, demonstrated an optimal method for isolation of foliar endophytes from both the plants. Our findings are in accordance with an earlier report which confirmed that the optimal concentration of sterilizing agent and time of exposure is the key foundation for isolation of a significant number of endophytes [22]. We were unable to isolate any endophyte when leaf samples were treated by employing the method 10 of sterilization (4% Sodium hypochlorite for 2 min and 99% EtOH for 30 s). It seems that the plant tissue and thus the endophytes might have been destroyed by this method of surface sterilization, which is an established fact cited in previous literature [22]. However, in your study, we haven’t verified this statement.

The colonization and isolation rate of fungal endophytes in the current study was well in the range of many host plants studied in the tropics [7, 27]. *Colletotrichum*, *Phomopsis*, *Fusarium*, *Chaetomium*, *Acremonium*, *Aspergillus*, and *Cladosporium* and *Xylaria* sp., which were frequently isolated from both the plants in this study, have been previously reported as endophytes in a varied host range in the tropics [23, 28].

The host-endophyte relationship varies from host to host and also depends on environmental conditions [29]. Although most of the species isolated were common in both the plants, but few species were selective to single host only. In this study, the diversity indices (H, J, and D) showed that the endophytic diversity was higher in *H. indicus* as compared to *A. racemosus*. Our results revealed that foliar endophyte assemblage of *H. indicus* slightly differed from *A. racemosus*. The occurrence of certain endophytes like *Curvularia* sp, *Fusarium* sp.1, *Gibberella moniliformis*, *Gloeosporium* sp., *Nigrospora sphaerica*, *Syncephalastrum racemosum* and *Penicillium* sp., were isolated from *H. indicus* but not from *A. racemosus*. Likewise, species like *Apiosordaria otanii*, *Colletotrichum lindemuthianum*, *Geotrichum clavatum*, *Gibberella avenacea*, *Glomerella acutata*, *Hemicola* sp., *Hypoxylon fragiforme*, *Myrothecium verrucaria* and *Pestalotiopsis guepinii* were isolated from *A. racemosus* but not from *H. indicus.* These findings suggest that some fungal endophytes species are host specific under certain environmental conditions, but the majority of the endophytes in general, adore the wide host range. The endophytes isolated in this study have been previously reported from wide host range in the tropics and sub-tropics [28, 30]. Though Simpsons endophyte diversity index was higher in *H. indicus* (D = 0.8) than *A. racemosus* (D = 0.5), mean overall colonization and mean overall isolation rates were comparatively lesser in *H. indicus* than the latter. The endophytic diversity of *A. racemosus* possessed *Acremonium strictum* and *Colletotrichum* sp., as dominant genera, followed by *Paraphoma* sp, however, in *H. indicus*, the dominant genera isolated was *A. strictum*.

The mean overall colonization and isolation rate for the individual season, as well as the relative colonization densities, were higher in September-November season for both the plants. Huang et al. [31] carried an indexed diversity study in 29 Chinese tropical medicinal plants and concluded that the overall colonization and isolation rates were relatively higher in the wet periods than the other seasons of the year. Likewise, studies of Suryanarayan and Thennarasan [30] on foliar endophytes of *P. rubra* established that temporal changes do affect the endophyte communities within the host plant. These findings support our data where we also found mean overall colonization and isolation rate highest in wet season (September‒November). In both *A. racemosus* and *H. indicus* the endophyte diversity, isolation, and colonization rates were greater during the September‒November season rather than the other seasons of the year.

The study area gets recurrent rainfall from the northeast monsoon disturbances through the Bay of Bengal from (mid)–September to December. During this period the study area witness an average 253 mm rainfall, compared to other months which bear just an average rainfall of 45 mm. The increased rainfall in these months makes the locale wet and misty. These changes affect the various plant–microbe, plant–fungi, or plant–plant interaction, which eventually influence the ecological niche of a habitat. Our findings conclusively reveal that the wet season is the most economical period for endophyte isolation, however, this needs to be further elucidated on different ranges of host plants and ecological or geographical niches. Higher rate of precipitation experienced in wet season could be the possible cause for the higher endophyte isolation rate in the wet season as precipitation is the major factor that influence infection by foliar endophytes. Several reviews suggest a strong correlation between endophyte infection levels and cumulative precipitation [32] and this was further corroborated by various other studies [23, 33–35]. Leaves sampled during the wet season, harbored more endophytes than in dry seasons as leaves become fully matured with very little precipitation during the dry season. Previously Strobel [36] reported the higher colonization frequency of endophytes occurring in *H. indicus* during the wet seasons rather than the dry seasons. Additionally, during wet seasons, higher rainfall promotes the dispersion of fungal spores and the moderate temperature helps in the greater viability of these fungal propagules for successive colonization in plant tissues. These fungal spores detach from the host by raindrops and disperse in splash droplets. The mucilage surrounding splash-borne spores protects them from desiccation and loss of viability under unfavorable conditions [37]. Climate change may alter the degree of mutualism between plant and fungi that even changes the efficacy of transmission of the endophyte from mother to daughter plant. Additionally the effect of climatic conditions like relative humidity, temperature, rainfall, moisture, etc. influence upon the stomatal conductance and mesophyll conductance of leaves controlling transpiration rates and availability of CO_2_ which in turn impact on the colonization of foliar fungal endophytes [15]. Moricca and Ragazzi [17] showed that the type of interaction between an endophyte and a plant is controlled by the genes of both and modulated by the environment. Therefore, a concurrent study performed on the seasonal recurrence of these endophytes suggests how the environmental conditions like temperature, humidity or rainfall, impact upon the isolation and colonization of endophytes from the host plant.

Conclusively, the present study indicates that, upon employing the finest method of sterilization, fungal endophytes could be harbored throughout the year though at different isolation and colonization rates. As evidenced by our study, the environmental factors like temperature and amount rainfall, positively impact the distribution of fungal endophytes within the host plant. However, this inference needs to be further elucidated with multi-geographic location study so that a coherent and integrated datum is established. Further, this study indicates that these two plants support a wide continuum of endophytes bearing significant bioactive prospective, hence, potential endophyte could be screened and explored in the medical, industrial or agricultural arena. In addition, the endophytic populations of these plants identified in this study may be studied in detail with the secondary metabolite perspective which may help to understand the bioactive potential of these endophytes.

## Conclusions

Employing the finest method of sterilization, fungal endophytes were copiously isolated from the two study plants. Among all treatments, study samples treated with 0.2% HgCl_2_ and 75% EtOH for 30 s and 1 min, respectively, demonstrated optimal isolation of the endophytes.

Both the plants demonstrated an affluent diversity of fungal endophytes in all the study seasons, however, the September–November season harbored the maximum number of endophytes in both the plants viz *A. racemosus* and *H. indicus* at 95.1 and 77.0% mean isolation rates respectively. The genera of ascomycetous fungi being the highest harvested genera in our study, harvested some potentially important endophyte species like *Acremonium*, *Colletotrichum*, and *Fusarium*. The isolated continuum of endophytes in the present study could be employed for investigating secondary metabolites to understand their ecosystem and to establish their potential role as industrial products and therapeutic targets.
